# Identification of Positive Regulators of the Yeast Fps1 Glycerol Channel

**DOI:** 10.1371/journal.pgen.1000738

**Published:** 2009-11-26

**Authors:** Sara E. Beese, Takahiro Negishi, David E. Levin

**Affiliations:** 1 Department of Biochemistry and Molecular Biology, Bloomberg School of Public Health, The Johns Hopkins University, Baltimore, Maryland, United States of America; 2 Department of Molecular and Cell Biology, Boston University Goldman School of Dental Medicine, Boston, Massachusetts, United States of America; Stanford University School of Medicine, United States of America

## Abstract

The yeast Fps1 protein is an aquaglyceroporin that functions as the major facilitator of glycerol transport in response to changes in extracellular osmolarity. Although the High Osmolarity Glycerol pathway is thought to have a function in at least basal control of Fps1 activity, its mode of regulation is not understood. We describe the identification of a pair of positive regulators of the Fps1 glycerol channel, Rgc1 (Ypr115w) and Rgc2 (Ask10). An *rgc1/2*Δ mutant experiences cell wall stress that results from osmotic pressure associated with hyper-accumulation of glycerol. Accumulation of glycerol in the *rgc1/2*Δ mutant results from a defect in Fps1 activity as evidenced by suppression of the defect through Fps1 overexpression, failure to release glycerol upon hypo-osmotic shock, and resistance to arsenite, a toxic metalloid that enters the cell through Fps1. Regulation of Fps1 by Rgc1/2 appears to be indirect; however, evidence is presented supporting the view that Rgc1/2 regulate Fps1 channel activity, rather than its expression, folding, or localization. Rgc2 was phosphorylated in response to stresses that lead to regulation of Fps1. This stress-induced phosphorylation was partially dependent on the Hog1 MAPK. Hog1 was also required for basal phosphorylation of Rgc2, suggesting a mechanism by which Hog1 may regulate Fps1 indirectly.

## Author Summary

When challenged by changes in extracellular osmolarity, many fungal species regulate their intracellular glycerol concentration to modulate their internal osmotic pressure. Maintenance of osmotic homeostasis prevents either cellular collapse under hyper-osmotic stress or cell rupture under hypo-osmotic stress. In baker's yeast, the Fps1 glycerol channel functions as the main vent for glycerol. Proper regulation of Fps1 is critical to the maintenance of osmotic homeostasis. In this study, we identify a pair of proteins (Rgc1 and Rgc2) that function as positive regulators of Fps1 activity. Their absence results in hyper-accumulation of glycerol and consequent cell lysis due to impaired Fps1 channel activity. Additionally, we found that these glycerol channel regulators function between the Hog1 (High Osmolarity Glycerol response) signaling kinase and Fps1, defining a signaling pathway for control of glycerol efflux. Because members of the Rgc1/2 family are found among pathogenic fungal species, but not in humans, they represent potentially attractive targets for antifungal drug development.

## Introduction

Under conditions of high osmolarity stress, many fungal species, including *Saccharomyces cerevisiae*, maintain osmotic equilibrium by producing and retaining high concentrations of glycerol as a compatible solute [1],[2]. Intracellular glycerol concentration is regulated in *S. cerevisiae* in part by the plasma membrane aquaglyceroporin, Fps1 [3]–[5]. Increased external osmolarity induces Fps1 closure, whereas decreased osmolarity causes channel opening, both within seconds of the change in external osmolarity [5]. This channel is required for survival of a hypo-osmotic shock when yeast cells have to rapidly export glycerol to prevent bursting [3],[5], and is required for controlling turgor pressure during fusion of mating yeast cells [6]. The pathway responsible for regulation of Fps1 in response to changes in osmolarity has not been delineated, but appears to involve the Hog1 (High Osmolarity Glycerol response) MAP kinase [5],[7],[8]. Hog1 is activated in response to hyper-osmotic stress to mediate the biosynthesis of glycerol and perhaps its retention as well through inhibition of Fps1 channel activity. Although a *hog1*Δ mutant displays an elevated rate of glycerol uptake in the absence of osmotic stress, it is not impaired for Fps1 closure in response to hyper-osmotic stress [5], suggesting that Hog1 regulates the basal activity of Fps1.

Fps1 is regulated not only in response to changes in external osmolarity, but also by exposure to acetic acid [9], and in response to trivalent metalloids (e.g. arsenite and antimonite) [10],[11]. Both acetic acid and metalloids enter the cell through Fps1 and induce Hog1 activation. Fps1 is down-regulated by acetic acid treatment through ubiquitin-mediated endocytosis, which is triggered by its phosphorylation by Hog1 on Thr231 and Ser537 [9]. By contrast, metalloids down-regulate both the expression of *FPS1* and its channel activity [10].

We describe the identification of a pair of paralogous *S. cerevisiae* proteins, Ask10 and Ypr115w that are positive regulators of glycerol efflux through Fps1. The *ASK10* and *YPR115w* genes encode members of a family of pleckstrin homology (PH) domain proteins in yeast that includes Slm1 and Slm2 [12]. The Ask10 protein shares 41% sequence identity with its paralog Ypr115w. Although PH domains are known to bind phosphatidylinositides [13], the PH domains of Ask10 and Ypr115w are interrupted by long insertions, prompting the suggestion that they bind different ligands [12], or even serve as protein-binding domains [14].

The *ASK10* gene was suggested previously to play a role in cell wall biogenesis through its identification in a genetic screen for activators of the Skn7 transcriptional regulator (Activator of SKN7) [15], which has been reported to influence cell wall assembly and cell wall stress signaling [16]–[19]. Additionally, Ask10 has been reported to be a component of the Srb/Mediator complex of RNA polymerase II [20], which is required for repression of several stress responsive genes [21],[22]. In this context, Ask10 was implicated in oxidative stress-induced destruction of the Srb11 C-type cyclin [20].

There are no reports on the function of *YPR115w*, or on the consequences of mutations in both Ask10 and Ypr115w. In this study, we describe the behavior of an *ask10*Δ *ypr115w*Δ mutant, finding that it displays a cell lysis defect that results from hyper-accumulation of glycerol. We find further that a defect in the function of the Fps1 glycerol channel is responsible for the *ask10*Δ *ypr115w*Δ phenotype. For this reason, we have given the name *RGC1* (for Regulator of the Glycerol Channel) to *YPR115w* and suggest *RGC2* as an alternate name for *ASK10*. Because the fungal kingdom is replete with members of this family of proteins, but they are not represented in animal cells, Rgc1/2 orthologs represent potentially attractive antifungal drug targets.

## Results

### An *rgc1/2*Δ mutant experiences cell wall stress

**Figure 1:**
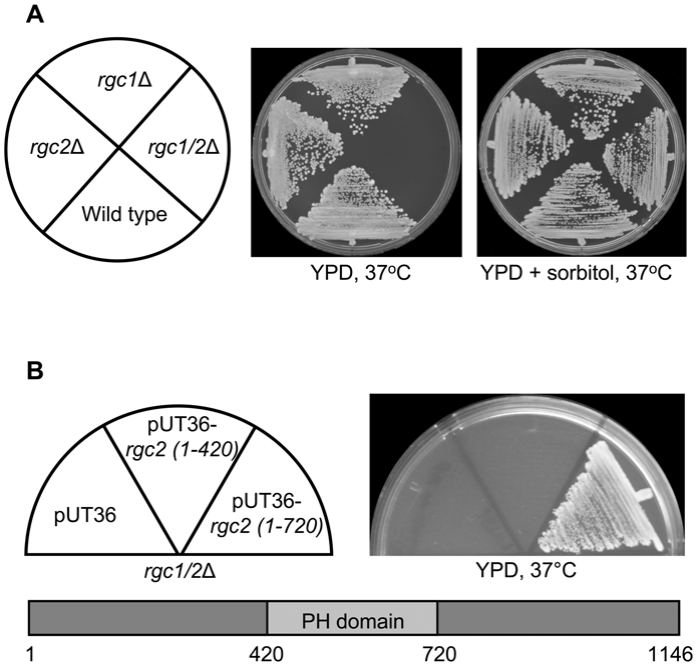
The *rgc1/2*Δ mutant displays an osmotic-remedial cell lysis defect. (A) Temperature sensitive cell lysis defect of the *rgc1/2*Δ mutant. Diploid yeast strains were streaked onto YPD plates with or without 10% sorbitol for osmotic support and incubated at 37°C for 3 days. Strains were wild-type (DL3193), *rgc2*Δ (DL3181), *rgc1*Δ (DL3203), and *rgc1/2*Δ (DL3209). (B) The PH domain of Rgc2 is important for its function. Yeast strain DL3209 was transformed with pUT36, pUT36 *MET25-rgc2 (1*–*420)-His_6_* (p2808), or pUT36 *MET25-rgc2 (1*–*720)-His_6_* (p2809). Transformants were streaked onto YPD plates and incubated at 37°C for 3 days. The schematic shows the PH domain relative to the truncations tested.

We constructed a double deletion mutant of *RGC1* and *RGC2* to test its susceptibility to cell wall stress. The double *rgc1/2*Δ mutant, but not the single mutants, displayed a temperature-sensitive growth defect (37°C; Figure 1A) accompanied by cell lysis, as judged by the presence of non-refractile “ghosts.” This result is in contrast to that reported by Cohen et al. [20], who found that deletion of *ASK10* (*RGC2*) alone conferred a temperature-sensitive phenotype in the same strain background. The growth defect of the *rgc1/2*Δ mutant was suppressed by inclusion of sorbitol in the medium for osmotic support (Figure 1A), indicating that cell lysis is the cause of the terminal mutant phenotype. To determine if the PH domain of Rgc2 was important for its function, we tested two C-terminal truncation mutants of *RGC2* for their ability to complement the *rgc1/2*Δ mutant cell lysis defect. The *rgc2 (1*–*720)* allele, which is missing the C-terminal 426 residues, but retains the PH domain, complemented the double mutant when over-expressed (Figure 1B). By contrast, the *rgc2 (1*–*420)* allele, which lacks the PH domain, failed to complement the double mutant. Neither allele complemented the mutant when expressed at low level from the chromosome (data not shown). This reveals that the C-terminal 426 residues are not critical to the function of Rgc2, and suggests that the PH domain contributes to its function.

**Figure 2:**
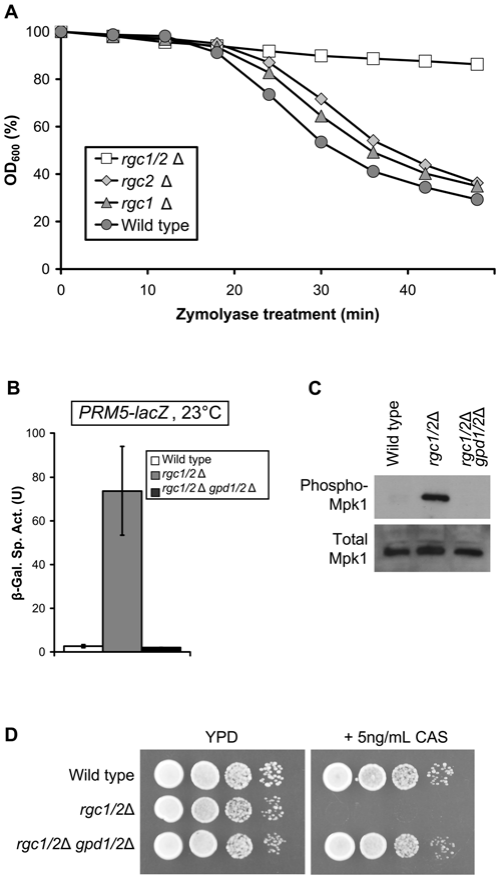
The *rgc1/2*Δ mutant experiences cell wall stress. (A) The *rgc1/2*Δ mutant is Zymolyase resistant. The strains from Figure 1A were grown to mid-log phase in YPD at 30°C, washed and resuspended in water to an initial density of A_600_ ∼ 0.7 prior to treatment with Zymolyase 20T (150 µg/ml). Cell lysis was assessed by A_600_ measurements at the indicated times. (B,C). The *rgc1/2*Δ mutant is under constitutive cell wall stress. (B) Wild-type yeast (DL3193), a *rgc1/2*Δ mutant (DL3209), and a *rgc1/2*Δ *gpd1/2*Δ mutant (DL3251) were transformed with a *PRM5-lacZ* (p1366) reporter plasmid to measure cell wall stress signaling. Transformants were grown to mid-log phase in selective medium at 23°C, followed by cell lysis and measurement of β-galactosidase activity. Each value represents the mean and standard deviation from three independent transformants. (C) Protein extracts from the strains in (B) were separated by SDS-PAGE and processed for immunoblot detection of activated Mpk1 (phospho-Mpk1) and total Mpk1 as a measure of cell wall stress signaling. (D) The *rgc1/2*Δ mutant is sensitized to caspofungin. The strains in (B) were grown to mid-log phase in YPD and 10-fold serial dilutions were spotted onto YPD plates with or without caspofungin (5 ng/ml) prior to incubation at 30°C for 3 days.

Mutants that display osmotic-remedial cell lysis are typically compromised for cell wall biogenesis. To test this, we measured the rate of cell lysis of the *rgc1/2*Δ mutant by digestion of the cell wall with zymolyase, a wall degrading enzyme. Surprisingly, this mutant did not lyse more rapidly than the wild-type strain, but displayed slower than normal lysis kinetics (Figure 2A), suggesting that it produces a fortified cell wall. The single *rgc1*Δ and *rgc2*Δ mutants were slightly more resistant to zymolyase than was wild-type. A mutant that produces a fortified cell wall, but is nevertheless susceptible to cell lysis upon imposition of a cell wall stress may be interpreted to be under constitutive cell wall stress. We tested this by measuring the transcriptional output of the cell wall integrity (CWI) pathway. The *rgc1/2*Δ mutant was strongly activated for transcription of a *PRM5-lacZ* reporter (Figure 2B), which is under the control of the Mpk1 MAP kinase and the Rlm1 transcription factor [23]. This mutant also displayed constitutively active Mpk1, as judged by the phosphorylation state of this MAP kinase (Figure 2C). These results confirm that the *rgc1/2*Δ mutant experiences severe cell wall stress, to which it responds by fortifying the cell wall, and also explains its lysis defect in response to additional cell wall stress at high temperature. In further support of this conclusion, we found that the *rgc1/2*Δ mutant is sensitized to growth inhibition by caspofungin (Figure 2D), an antifungal drug that interferes with cell wall biosynthesis by inhibiting β-glucan synthase activity [24]. Caspofungin treatment prevents the fortification of the cell wall that is essential to the survival of this mutant.

### Rgc1 and Rgc2 serve a redundant role in the regulation of glycerol efflux through the Fps1 glycerol channel

**Figure 3:**
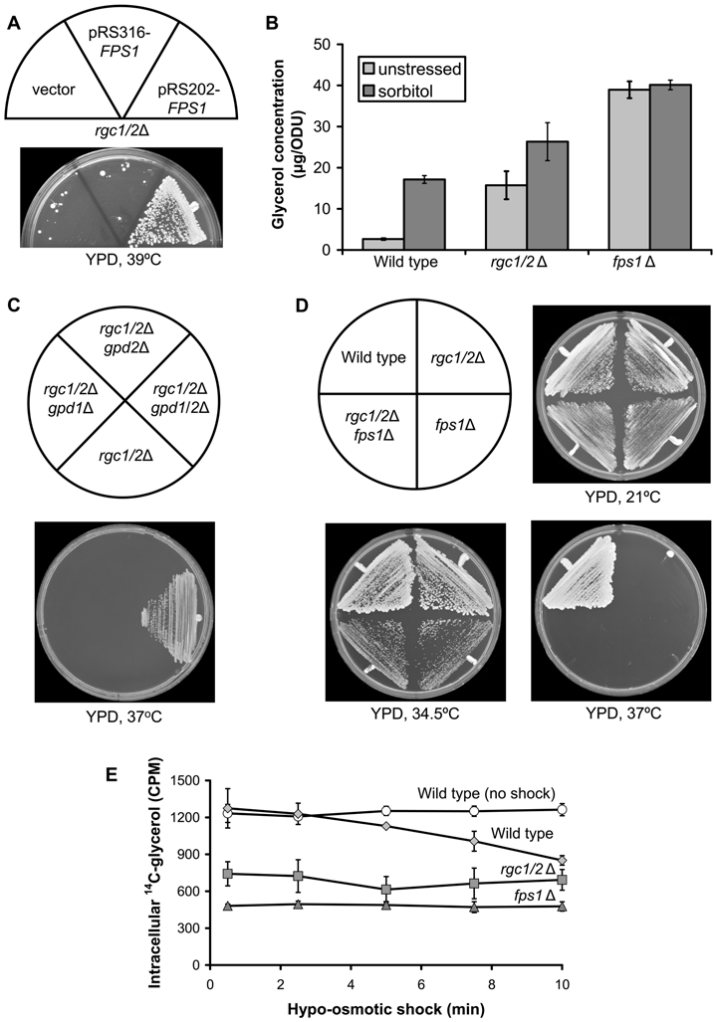
The *rgc1/2*Δ mutant is defective for glycerol efflux through Fps1. (A) Suppression of the cell lysis defect of the *rgc1/2*Δ mutant by overexpressed *FPS1*. A *rgc1/2*Δ mutant (DL3209) was transformed with centromeric, or high-copy plasmids bearing *FPS1* (pRS316-*FPS1* or pRS202-*FPS1*, respectively), or vector (pRS316). Transformants were streaked onto a YPD plate and incubated for 3 days at 39°C. (B) Intracellular glycerol concentrations in wild-type (DL3193), *rgc1/2*Δ (DL3209), and *fps1*Δ (DL3234) strains. Cultures were grown to mid-log phase in YPD, diluted into YPD with or without sorbitol (to 1.8M) to induce hyper-osmotic shock (15 minutes). Each value represents the mean and standard deviation from three independent experiments. (C) The cell lysis defect of the *rgc1/2*Δ mutant is suppressed by blocking glycerol biosynthesis. Diploid yeast strains were streaked onto a YPD plate and incubated at 37°C for 3 days. Strains were: *rgc1/2*Δ (DL3209), *rgc1/2*Δ *gpd1*Δ (DL3237), *rgc1/2*Δ *gpd2*Δ (DL3254) and *rgc1/2*Δ *gpd1/2*Δ (DL3251). (D) The cell lysis defect of the *rgc1/2*Δ mutant is not additive with that of an *fps1*Δ mutant. Diploid yeast strains were streaked onto YPD plates and incubated for 3 days at the indicated temperatures. Strains were: wild-type (DL3193), *rgc1/2*Δ (DL3209), *fps1*Δ (DL3234), and *rgc1/2*Δ *fps1*Δ (DL3245). (E) The *rgc1/2*Δ mutant is blocked for glycerol efflux. Cells were pre-loaded with ^14^C-labelled glycerol (MES buffer with 300 mM glycerol), followed by hypo-osmotic shock (into MES buffer) for the indicated times. Strains were wild-type (DL3193), *rgc1/2*Δ (DL3209), and *fps1*Δ (DL3234). Each value represents the mean and standard deviation from three independent experiments.

To understand the cause of the cell wall stress in the *rgc1/2*Δ mutant, we conducted a dosage suppressor screen for high-copy number plasmids that conferred growth at 37°C. A single class of strong suppressor was identified as the *FPS1* gene (Figure 3A). *FPS1* encodes an aquaglyceroporin that is the major facilitator of glycerol uptake and efflux in yeast [3],[5]. This plasma membrane channel protein also mediates uptake of toxic metalloids, such as arsenite and antimonite [10],[11]. One interpretation of the suppression result is that the *rgc1/2*Δ mutant experiences abnormally high turgor pressure from accumulation of glycerol, which yeast cells use as a compatible solute for osmoregulation. Measurement of intracellular glycerol concentration confirmed that the *rgc1/2*Δ mutant has a 5.9-fold higher glycerol level than wild-type cells under normal growth conditions, a value that is approximately half that of an *fps1*Δ mutant and approximately equal to that of wild-type cells exposed to hyper-osmotic shock (Figure 3B). To determine if excess intracellular glycerol is responsible for the phenotypic defects of this mutant, we blocked glycerol biosynthesis at the first committed and rate limiting step, glycerol-3-phosphate dehydrogenase (GPD) [25]–[27]. GPD is encoded by the paralogous genes *GPD1* and *GPD2*. Deletion of either *GPD1* or *GPD2* alone did not suppress the lysis defect of the *rgc1/2*Δ mutant, but blocking glycerol biosynthesis completely by deletion of both *GPD1* and *GPD2* allowed growth at 37°C (Figure 3C), confirming that glycerol accumulation is responsible for the cell lysis defect. This also provides an explanation for the fortified cell wall of the *rgc1/2*Δ mutant as a response to the stress of abnormally high turgor pressure. Consistent with this interpretation, the *gpd1/2*Δ mutations relieved the cell wall stress signaling observed in the *rgc1/2*Δ mutant (Figure 2B and 2C). Finally, the *gpd1/2*Δ mutations relieved the caspofungin sensitivity of the *rgc1/2*Δ mutant (Figure 2D).

We considered two possible explanations for the hyper-accumulation of glycerol in the *rgc1/2*Δ mutant – the mutant either produces excess glycerol, or it is impaired for glycerol efflux through Fps1. These hypotheses generate different predictions for the impact of the *rgc1/2*Δ mutations on the phenotype of an *fps1*Δ mutant. If the *rgc1/2*Δ mutant produces excess glycerol, this should exacerbate the lysis defect of an *fps1*Δ mutant, which is blocked for glycerol efflux. By contrast, if the *rgc1/2*Δ mutant is blocked for glycerol efflux through Fps1, loss of the glycerol channel should not result in an additive defect. Figure 3D shows that an *fps1*Δ mutant displays a temperature-sensitive growth defect that is slightly more severe than that of the *rgc1/2*Δ mutant, with a semi-permissive growth temperature of 34.5°C. The *fps1*Δ mutant growth defect at elevated temperature is also the result of cell lysis (data not shown). Significantly, the *rgc1/2*Δ *fps1*Δ triple mutant behaves identically to the *fps1*Δ mutant (Figure 3D), supporting the hypothesis that the *rgc1/*2Δ mutant is impaired for glycerol efflux through Fps1. To test this directly, we measured export of glycerol from cells exposed to a hypo-osmotic shock, a condition that would induce glycerol efflux through Fps1. We found that glycerol was released from wild-type cells, but not from the *rgc1/2*Δ mutant or the *fps1*Δ mutant (Figure 3E), supporting the conclusion that Rgc1 and Rgc2 regulate glycerol efflux through Fps1. The varied initial content of ^14^C-labeled glycerol among these mutants is a consequence of differential glycerol loading, reflecting the importance of Fps1 for glycerol influx as well as efflux. Finally, strong overexpression of *RGC2* failed to suppress the temperature-sensitivity of the *fps1*Δ mutant (data not shown), thus establishing an epistatic relationship that places *RGC1* and *RGC2* above *FPS1* in a common pathway for glycerol efflux.

**Figure 4:**
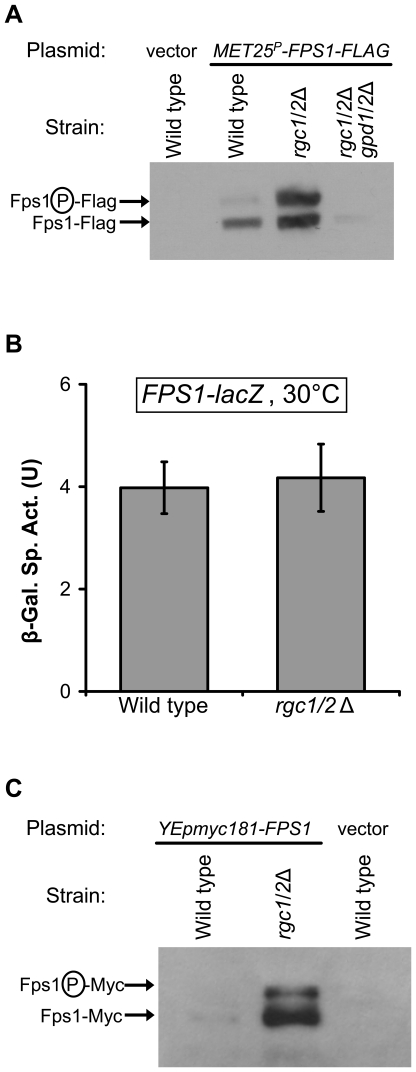
The *rgc1/2*Δ mutant retains elevated levels of the Fps1 protein resulting from excess intracellular glycerol. (A) Yeast strains were transformed with a plasmid expressing Fps1-FLAG under the control of the *MET25* promoter (p2492), or empty vector. Extracts were prepared from cultures grown in selective medium, protein was separated by SDS-PAGE, and Fps1-FLAG was detected by immunoblot analysis using a mouse monoclonal α-FLAG antibody. Strains were: wild-type (DL3193), *rgc1/2*Δ (DL3209), and *rgc1/2*Δ *gpd1/2*Δ (DL3251). (B) *FPS1-lacZ* transcription is not altered in the *rgc1/2*Δ mutant. An *FPS1-lacZ* reporter plasmid (p2213) was transformed into a wild-type strain (DL3193) and a *rgc1/2*Δ mutant (DL3209). Transformants were grown to mid-log phase in selective medium at 30°C, followed by cell lysis and measurement of β-galactosidase activity. Each value represents the mean and standard deviation from three independent transformants. (C) A greater increase in Fps1 protein levels is seen when *FPS1* is expressed from its native promoter. Yeast strains were transformed with a 2-micron plasmid expressing Fps1-Myc under the control of its own promoter (p2184), or empty vector. Extracts were treated as above, and Fps1-Myc was detected by immunoblot analysis using a mouse monoclonal α-Myc antibody. Strains were: wild-type (DL3193) and *rgc1/2*Δ (DL3209).

To explore the mechanism by which Rgc1/2 regulate Fps1, we first examined the Fps1 protein level in an *rgc1/2*Δ mutant. Despite the observed defect in glycerol efflux of the *rgc1/2*Δ mutant, this mutant maintains strongly elevated Fps1 protein levels as compared to wild-type (increased approximately 10-fold; Figure 4A), suggesting that it attempts to compensate for impaired Fps1 function by increasing the number of channel proteins. The increase in Fps1 protein is a consequence of elevated glycerol concentration resulting from the *rgc1/2*Δ mutation, because the Fps1 level was reduced in an *rgc1/2*Δ *gpd1/2*Δ mutant (Figure 4A), which is blocked for glycerol production. The increased steady-state level of Fps1 in the *rgc1/2*Δ mutant is not the result of transcriptional induction, because *FPS1* was expressed under the control of a heterologous promoter (*MET25*). This conclusion was supported by the finding that expression from an *FPS1-lacZ* reporter was not altered in the *rgc1/2*Δ mutant (Figure 4B). An even greater increase in Fps1 protein level in the *rgc1/2*Δ compared to wild-type (approximately 20-fold) was observed when *FPS1* was expressed from its native promoter on a multi-copy plasmid (Figure 4C). Evidently, ectopic overexpression of *FPS1* suppresses the *rgc1/2*Δ lysis defect by assisting the cell in its efforts to enhance glycerol efflux through an impaired channel. Under these conditions the cells retain more than 20-fold higher levels of Fps1 protein than wild-type cells (the comparison in Figure 4C was to wild-type cells also expressing *FPS1* from a multi-copy plasmid). Therefore, we conclude that Fps1 channels in the *rgc1/2*Δ mutant retain less than 5% of normal activity.

To determine the cause of the increased steady-state level of Fps1 in the *rgc1/2*Δ mutant, we conducted a test of Fps1 stability. Fps1 levels were followed in cells in which *FPS1* transcription was shut down with the simultaneous inhibition of protein synthesis. We found that Fps1 was stabilized in the *rgc1/2*Δ mutant relative to wild-type cells (Figure S1). Therefore, we conclude that increased intracellular glycerol in the *rgc1/2*Δ mutant, which is caused by a deficiency in Fps1 function, induces an increase in the level of weakly functional Fps1 through protein stabilization.

We conclude further that, because the *rgc1/2*Δ mutant does not display diminished Fps1 levels, Rgc1/2 positively regulate Fps1 function by a mechanism other than increased protein level. Fps1 migrates as a doublet as a consequence of phosphorylation [11], although the responsible protein kinase has not been identified. It is interesting to note that the more slowly migrating band (the phosphorylated form) predominates in the *rgc1/2*Δ mutant (Figure 4A).

**Figure 5:**
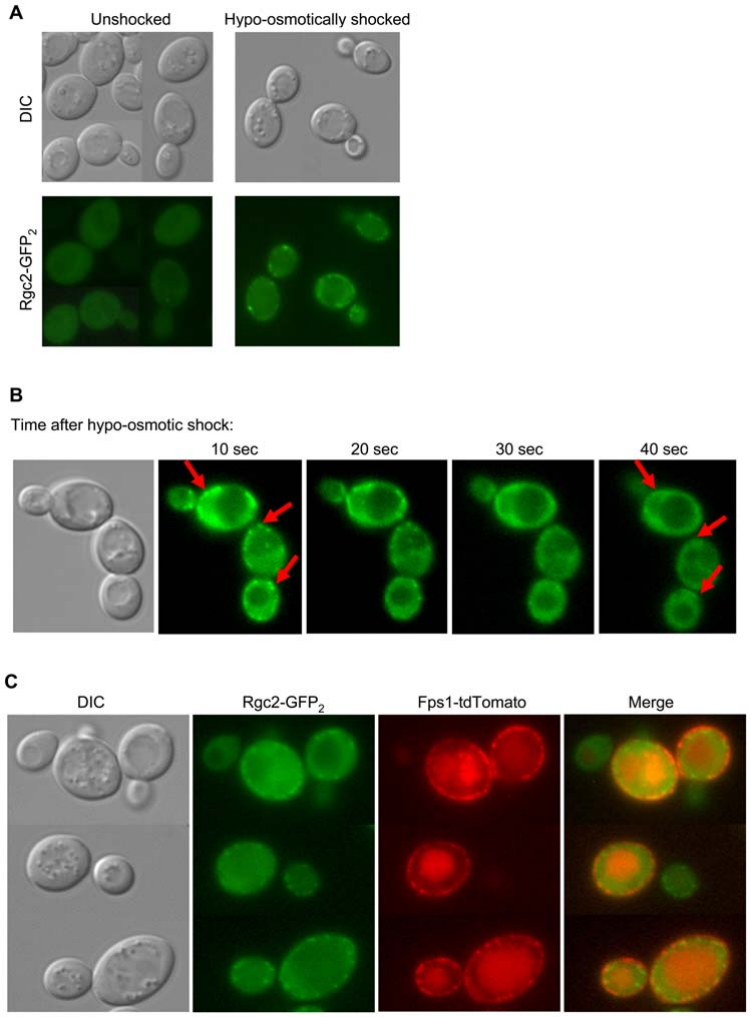
Localization of Rgc2. (A) Rgc2-GFP_2_ re-localizes from uniform cytoplasmic distribution to punctate spots near the cell periphery in response to hypo-osmotic shock. Wild-type diploid yeast cells (DL3193), transformed with a plasmid expressing Rgc2-GFP_2_ (p2481), were grown to mid-log phase in SD medium, centrifuged briefly, and resuspended in distilled water to induce hypo-osmotic shock. Shocked cells were mounted for fluorescence microscopy and photographed within 10 seconds of shock. (B) Dissipation of Rgc2-GFP_2_ spots after hypo-osmotic shock. Cells were treated as in (A) and photographed at the indicated times after shock. (C) The punctate spots of Rgc2-GFP_2_ do not co-localize with those of Fps1-tdTomato. Wild-type cells (DL3193), co-transformed with p2481 and a plasmid expressing Fps1-tdTomato (p2489), were subjected to hypo-osmotic shock for 10 seconds and examined for co-localization of Rgc2 with Fps1.

Both Rgc1 and Rgc2 have been reported to reside in the cytoplasm [28]. If these proteins function as activators of the Fps1 glycerol channel, they might be expected to interact with Fps1 at the plasma membrane. We examined the intracellular localization of Rgc2-GFP_2_ in response to hypo-osmotic shock, conditions in which the Fps1 channel must be opened to allow glycerol efflux. Figure 5A shows that under unstressed conditions, Rgc2-GFP_2_ displays diffuse cytoplasmic localization, but very rapidly aggregates into punctate spots that appear near the cell surface in response to hypo-osmotic shock. These spots dissipate over a period of approximately 45 seconds (Figure 5B). Fps1 has been reported to reside in punctate spots at the plasma membrane [5]. Therefore, we asked if Rgc2-GFP_2_ co-localizes with Fps1-tdTomato in response to hypo-osmotic shock. Figure 5C shows that these spots do not co-localize. Other efforts to detect physical interaction between Rgc2 and Fps1 (e.g. co-precipitation and two-hybrid analyses; data not shown) failed to provide such evidence. Additionally, the number, location, and intensity of Fps1 punctate spots do not appear to be altered in an *rgc1/2*Δ mutant (Figure S2). This last result is difficult to understand considering that the mutant retains much more Fps1. It is possible that the fluorescent protein is preferentially cleaved from the stabilized Fps1 and digested in the vacuole. Nevertheless, the Fps1 we can detect in the *rgc1/2*Δ mutant appears to reside in the same location as in wild-type cells. These results, taken in the aggregate, suggest that regulation of Fps1 by Rgc1/2 is at the level of channel activity, rather than channel expression or localization.

### Open channel mutants of Fps1 retain their open channel character in the absence of Rgc1/2

**Figure 6:**
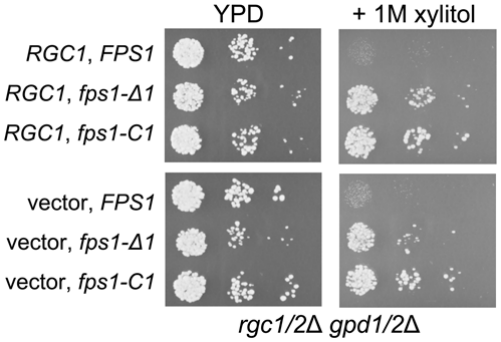
Growth in the presence of 1M xylitol as a test of open channel behavior of *fps1* alleles. An *rgc1/2*Δ mutant that was also blocked for glycerol production (*gpd1/2*Δ; DL3246) was co-transformed with multi-copy plasmids bearing wild-type or the indicated open channel alleles of *FPS1* and a centromeric plasmid bearing *RGC1* (p2627), or a vector control (pRS313). Transformants were grown to mid-log phase in selective medium and 10-fold serial dilutions were spotted onto YPD plates with or without 1M xylitol prior to incubation at 30°C for 3 days or 2 days, respectively.

Fps1 is a multi-pass plasma membrane protein with cytoplasmic N-terminal and C-terminal extensions that are inhibitory to channel function [5],[29],[30]. Truncation of these extensions results in constitutively open forms of the Fps1 channel. To explore the dependence of open channel character of Fps1 mutants on Rgc1/2 function, we tested their ability to allow xylitol uptake. A *gpd1/2*Δ mutant is very sensitive to high external osmolarity, because it cannot produce glycerol to re-establish osmotic balance. However, open channel *fps1* mutants suppress this defect when hyper-osmotic shock is induced by 1M xylitol, which enters the cell only through unregulated Fps1 to restore osmotic balance [30]. Although a *gpd1/2*Δ mutant expressing wild-type *FPS1* grew very poorly in the presence of xylitol, two Fps1 open channel mutants, one with an N-terminal truncation (*fps1-*Δ*1*, produces Fps1^Δ12–231^) [5], the other with a C-terminal truncation (*fps1-C 1* produces Fps1^Δ534–650^) [30], conferred growth on xylitol to a *gpd1/2*Δ mutant even in the absence of *RGC1/2* (Figure 6). This result indicates that the open channel mutants of Fps1 obviate the requirement for Rgc1/2 for Fps1 function, and support the conclusion that Fps1 is properly folded and localized independently of Rgc1/2 function.

### Mutations in *RGC1* and *RGC2* confer resistance to the toxic metalloid arsenite by blocking Fps1 function

**Figure 7:**
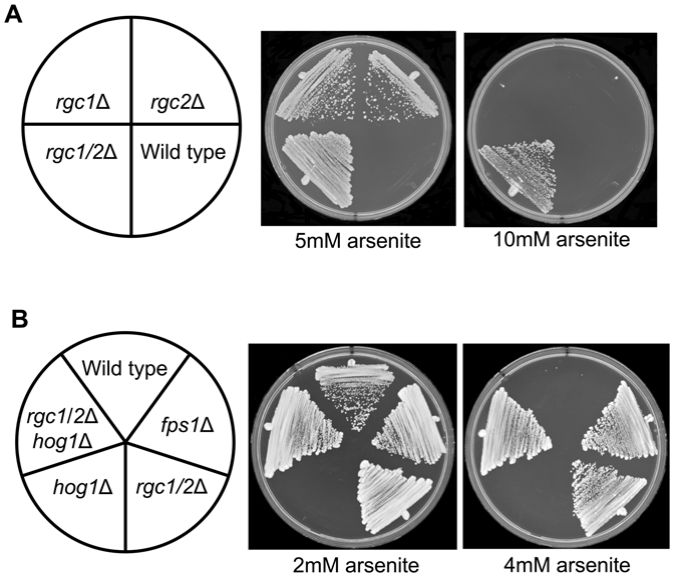
Mutants blocked for Fps1 function are resistant to arsenite toxicity. (A) Diploid yeast strains were streaked onto YPD plates with the indicated concentration of arsenite and incubated at 30°C for 3 days. Diploid yeast strains were wild-type (DL3193), *rgc1*Δ (DL3203), *rgc2*Δ (DL3181), and *rgc1/2*Δ (DL3209). (B) The *rgc1/2*Δ mutations suppress the arsenite hyper-sensitivity of a *hog1*Δ mutant. Haploid yeast strains were wild-type (DL3187), *fps1*Δ (DL3226), *rgc1/2*Δ (DL3207), *hog1*Δ (DL3158), and *rgc1/2*Δ *hog1*Δ (DL3219).

The toxic metalloids arsenite and antimonite enter yeast cells through the Fps1 channel [10],[11]. An *fps1*Δ mutant is therefore resistant to toxicity of these metalloids. As a further test of the role of Rgc1 and Rgc2 in the regulation of Fps1, we examined the sensitivity of mutants in these genes to arsenite. Wild-type cells were sensitive to growth inhibition by 5 mM arsenite, but both the *rgc1*Δ and *rgc2*Δ mutants were resistant to this treatment (Figure 7A). Moreover, the *rgc1/2*Δ double mutant was resistant to 10 mM arsenite, consistent with the additive nature of Rgc1 and Rgc2 function. These results further support the conclusion that Rgc1/2 function is required to open Fps1. Thorsen et al. [11] demonstrated that the Hog1 MAP kinase is activated in response to arsenite treatment and that Hog1 is required for control of basal Fps1 channel activity. A *hog1*Δ mutant was shown to display increased arsenite uptake and hyper-sensitivity to arsenite toxicity, both phenotypes being blocked by an *fps1*Δ mutation. Therefore, to place Hog1 within the Rgc1/2 – Fps1 pathway, we tested an *rgc1/2*Δ *hog1*Δ triple mutant for arsenite sensitivity. Like the *rgc1/2*Δ mutant, the *rgc1/2*Δ *hog1*Δ mutant was resistant to arsenite toxicity (Figure 7B). Suppression of the *hog1*Δ arsenite hyper-sensitivity defect by the *rgc1/2*Δ mutations indicated that Fps1 is closed in the triple mutant. These results suggest that Hog1 promotes Fps1 closure by inhibiting the action of Rgc1/2. The order of function of these pathway components was supported by the observation that the cell lysis defect of the *rgc1/2*Δ mutant was not suppressed by the *hog1*Δ mutation (data not shown).

### Rgc2 is phosphorylated in response to various stresses

**Figure 8:**
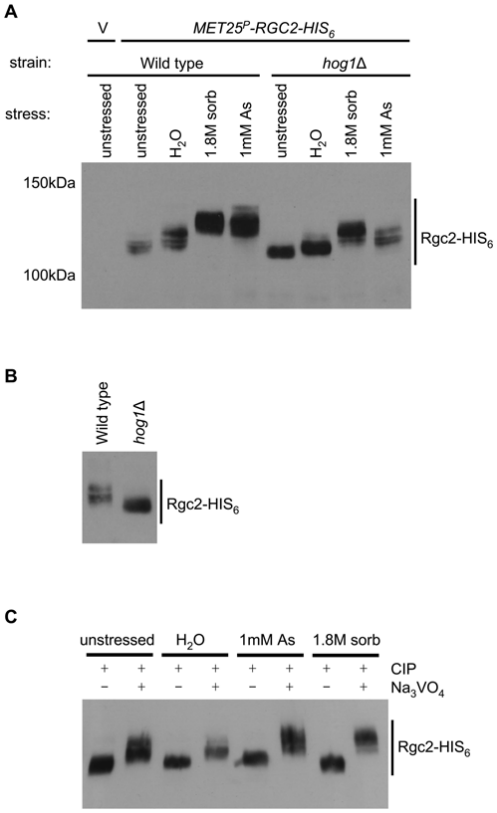
Stress-induced phosphorylation of Rgc2. (A) Stresses induce a band-shift in Rgc2 that is only partially dependent on Hog1. Wild-type (DL3187) or *hog1*Δ (DL3158) cells, transformed either with a plasmid that expresses Rgc2-His_6_ (p2501), or vector control (V, pUT36), were treated with stresses that cause Fps1 opening (hypo-osmotic shock; H_2_O), or closure (hyper-osmotic shock, or arsenite). Hypo-osmotic shock and hyper-osmotic (1.8M sorbitol) shock were for 1 minute, and arsenite (As) treatment was for 1 hour. Protein extracts were prepared and separated by SDS-PAGE for immunoblot detection of Rgc2-His_6_. (B) The unstressed samples from (A) were run side-by-side to illustrate the Hog1-dependent band-shift of Rgc2-His_6_. (C) Rgc2 band-shifts are caused by phosphorylation. Rgc2-His_6_ was immuneprecipitated from extracts of wild-type (DL3187) cells treated as above, and subjected to calf intestinal phosphatase (CIP) treatment in the presence or absence of phosphatase inhibitor (Na_3_VO_4_). Immuneprecipitates were processed for immunoblot detection of Rgc2-His_6_.

Because epistasis analysis revealed that Hog1 acts upstream of Rgc1 and Rgc2 to oppose their function, we asked if Rgc2 becomes phosphorylated in response to stresses that lead to the opening or closing of the Fps1 channel. Cells expressing C-terminally His-tagged Rgc2 were subjected to hypo-osmotic shock, hyper-osmotic shock (with sorbitol), or arsenite treatment. Rgc2 displayed mobility shifts on SDS-PAGE in response to all three of these stresses (Figure 8A), presumably reflecting post-translational modifications. The treatments that result in Fps1 closure (arsenite and hyper-osmotic shock) induced the greatest shifts, but hypo-osmotic shock, which induces Fps1 opening, also caused a detectable band-shift. In fact, multiple bands were detectable even in Rgc2 from unstressed cells. To determine if these mobility shifts were dependent upon Hog1, we examined Rgc2 mobility in a *hog1*Δ mutant. The absence of Hog1 did not prevent the stress-induced Rgc2 band-shifts, but in all cases reduced the extent of shift (Figure 8A). Rgc2 from unstressed cells also displayed increased mobility in a *hog1*Δ mutant (Figure 8B), suggesting that Rgc2 sustains a basal level of Hog1-dependent phosphorylation.

This experiment also revealed the existence of additional modifications in response to these stresses that are Hog1-independent. To determine if these additional modifications were phosphorylations, we subjected Rgc2 isolated from stressed cells to protein phosphatase treatment. For all three stresses, phosphatase treatment collapsed the Rgc2 band to the same level as phosphatase treated, unstressed Rgc2 (Figure 8C). We conclude that although basal phosphorylation of Rgc2 is Hog1-dependent, other protein kinases are responsible for the hyper-phosphorylation observed in response to Fps1-regulating stresses.

It has been demonstrated that in the absence of Hog1, hyper-osmotic stress activates the Fus3 and Kss1 MAP kinases through inappropriate cross-talk [31]. Therefore, to determine if the Rgc2 band-shift observed in response to high osmolarity in the absence of Hog1 was due to such cross-talk, we tested a *hog1*Δ *ste11*Δ double mutant, which is blocked for activation of Fus3 and Kss1. The mobility shift observed for Rgc2 in this mutant was indistinguishable from that of the *hog1*Δ mutant (Figure S3), indicating that these MAP kinases are not responsible for the hyper-osmotic stress-induced phosphorylation.

## Discussion

Glycerol serves as a compatible solute in *S. cerevisiae* and other yeasts, allowing cells to respond quickly to changes in external osmolarity. A key component in the control of cytoplasmic glycerol concentration is the Fps1 glycerol channel. Although Fps1 is known to close under conditions of hyper-osmotic stress, and open in response to hypo-osmotic shock [3],[5], the mechanism by which Fps1 function is modulated is not understood. In this study, we describe a regulatory pathway for the control of this glycerol channel.

### Glycerol channel regulatory proteins

We identified a pair of paralogous genes, *RGC1* (Regulator of the Glycerol Channel; *YPR115w*) and *RGC2* (*ASK10*), that function as positive regulators of Fps1. The studies described reveal that loss of function of both *RGC1/2* results in cell wall stress that is caused by excess turgor pressure associated with elevated intracellular glycerol concentration. The increase in glycerol is the consequence of impaired Fps1 function. We found that the increased turgor pressure experienced by the *rgc1/2*Δ mutant provokes the cell to activate the CWI signaling pathway and to fortify the cell wall. Nevertheless, imposing additional cell wall stress on this mutant induced cell lysis, a defect that was suppressed by blocking glycerol synthesis. In this regard, it is interesting to note that blocking the function of the glycerol channel activators also sensitized cells to caspofungin, an antifungal agent that acts by inhibiting cell wall biosynthesis [24]. Evidently, preventing the cells from responding to their internally imposed cell wall stress is lethal. Therefore, Rgc1/2 might be suitable antifungal targets for combination therapy with caspofungin.

The mechanism by which Rgc1/2 regulate Fps1 remains unclear. Although there is some evidence that Rgc2 (Ask10) can act as a transcriptional regulator (see below), we did not find that Rgc1/2 control Fps1 transcription. We were not able to detect direct interaction between Rgc2 and the Fps1 channel. However, the findings that Fps1 localizes to the plasma membrane in the presence or absence of Rgc1/2 and that constitutive mutants of Fps1 retain their open channel character independently of Rgc1/2 suggests that these proteins regulate Fps1 through its activity, rather than at an earlier step, such as protein folding, or proper localization. Rgc1/2 control of Fps1 folding or localization would be expected to impact the function of open channel mutants as well as the wild-type.

### The role of Hog1 in the regulation of Fps1

Fps1 is unusual in its possession of extensions at both its cytoplasmic N-terminus and C-terminus that play a role in regulating Fps1 channel activity [29],[30]. These extensions have been suggested to function as flaps that restrict the flow of glycerol through the channel. However, the mechanism by which they respond to changes in extracellular osmolarity remains largely unknown.

The HOG pathway is activated in response to hyper-osmotic stress [8]. Hog1, the stress-activated MAP kinase at the base of this pathway plays a poorly-defined role in the regulation of Fps1. A *hog1*Δ mutant exhibits a glycerol uptake rate that is approximately 3-fold-higher than that of wild-type cells [5],[11]. However, this mutant is not impaired for Fps1 closure in response to hyper-osmotic stress [5], suggesting that Hog1 regulates the basal activity of Fps1 (i.e. in the absence of osmostress), but not the osmotic stress-induced closure. Basal inhibition of Fps1 by Hog1 may result from phosphorylation at Thr231, which resides within the N-terminal extension, because Hog1 can phosphorylate this site in vitro [11], and mutation of Thr231 to Ala results in constitutive Fps1 activity [11],[29].

In addition to glycerol, the toxic metalloid arsenite enters the cell through the Fps1 glycerol channel [10]. Loss of Fps1 function confers resistance to arsenite, whereas loss of Hog1 function results in an increase in the rate of arsenite uptake through Fps1 and consequent hyper-sensitivity to the metalloid [11]. We found that null mutations in *RGC1/2* also conferred resistance to arsenite, consistent with the conclusion that Rgc1 and Rgc2 are important for Fps1 channel activity. The *rgc1/2*Δ mutations suppressed the arsenite hyper-sensitivity of a *hog1*Δ mutation. In fact, loss of *RGC1/2* function was completely epistatic to the *hog1*Δ mutation with regard to arsenite sensitivity, suggesting that Hog1 exerts its negative effect on Fps1 channel function by inhibiting Rgc1 and Rgc2.

We found that Rgc2 undergoes phosphorylation-induced band-shifts in response to various Fps1-regulatory stresses (i.e. hypo- and hyper-osmotic shock, and arsenite stress). These phosphorylations were partially dependent on Hog1, as intermediate shifts were observed in a *hog1*Δ mutant. Rgc2 also appears to undergo basal phosphorylation that is Hog1-dependent. The PhosphoPep database (part of the *Saccharomyces* Genome Database) [32] identifies 5 phosphorylation sites on Rgc1 and 10 in Rgc2 from unstressed cells. However, only one of these sites in Rgc2 (Thr808), and none in Rgc1 reside at potential Hog1 phosphorylation motifs (S/TP), suggesting that the observed Hog1-basal phosphorylation of Rgc2 is largely, or entirely indirect.

It is also possible that Hog1 inhibits basal Fps1 activity both directly, through phosphorylation of Thr231, and indirectly through phosphorylation of Rgc1/2. In any case, it is clear that other protein kinases contribute to the regulation of Rgc2 (and probably Rgc1), and consequently Fps1, in response to various stresses. These results establish a regulatory pathway from Hog1 to Rgc1/2 to Fps1, in which Rgc1 and Rgc2 are positive regulators of Fps1 channel activity and Hog1 inhibits Fps1 through inhibition of Rgc1/2. Although the interaction between these proteins and Hog1 may be direct, the phosphorylation sites on Rgc1 and Rgc2 remain to be identified.

### Other functions of Rgc2 (Ask10)

It is possible that Rgc1/2 are multifunctional proteins. Overexpression of Ask10 was reported to enhance growth of a strain in which histidine production was under the control of (*lexA_op_*)-*HIS3* reporter driven by a LexA-Skn7 fusion [15]. However, *ASK10* overexpression failed to drive a similarly regulated (*lexA_op_*)-*lacZ* reporter. This was in contrast to the behavior of *MID2*, another gene identified in this screen that activated both reporters [18], raising the possibility that Ask10 does not activate Skn7-mediated transcription.

A second report, by Cohen et al. [20], suggested that Ask10 participates in the oxidative stress-induced destruction of Srb11, a C-type cyclin that is part of the Mediator complex of RNA polymerase II. These investigators identified Ask10 in a two-hybrid screen for Srb11-interacting proteins. They further demonstrated that, like Srb11 and its cyclin-dependent kinase (Srb10), Ask10 is a component of the RNA polymerase II holoenzyme. We do not know how the function of Rgc1/2 as regulators of Fps1 might relate to their reported roles in stress-activated transcription.

Rgc1 and Rgc2 are large proteins (120kD and 127kDa, respectively), and our immunoblot analysis of Rgc2 suggests that its regulation in response to different stresses that regulate Fps1 is complex. The unstressed and stressed forms of Rgc2 all migrate as several distinct bands. We have shown that these bands represent a variety of phosphorylated states of Rgc2. Although identities of many of the phosphorylation sites are not known, numerous Rgc1 and Rgc2 phosphorylation sites have been identified in response to DNA damage stress. Albuquerque et al. [33] identified 17 phosphorylation sites in Rgc1 and 20 in Rgc2 in response to treatment with the DNA alkylating agent, MMS. Additionally, as noted above, numerous basal phosphorylation sites in Rgc1 and Rgc2 are reported the PhosphoPep database [32]. Only a few of these sites overlap with those found in MMS-treated cells.

Finally, Cohen et al. [20] found that Rgc2 (Ask10) is phosphorylated in response to oxidative stress induced by hydrogen peroxide. These authors reported that the redundant MAPK kinases of the Cell Wall Integrity (CWI) signaling pathway (Mkk1 and Mkk2) were responsible for this modification. Oddly, however, none of the four MAP kinases in yeast were found to be involved [20]. We revisited this result, finding that none of the kinases within the CWI MAPK cascade (including Mkk1/2) were required for the oxidative stress-induced phosphorylation of Rgc2 (Figure S4).

Rgc1/2 may function to integrate multiple stress signals, only some of which are known to control Fps1 channel activity. The regulation of Rgc1/2 by phosphorylation in response to different stresses appears to be complex. Moreover, these proteins may have additional functions that have yet to be identified.

## Materials and Methods

### Strains, growth conditions, and transformations

The *S. cerevisiae* strains used in this study are listed in Table 1. Yeast cultures were grown in YPD (1% Bacto yeast extract, 2% Bacto Peptone, 2% glucose) or SD (0.67% Yeast nitrogen base, 2% glucose) supplemented with the appropriate nutrients to select for plasmids. Yeast strains bearing multiple deleted genes were constructed by genetic crosses, followed by PCR-based detection of the deleted alleles. Diploid strains were used for most experiments, because the cell lysis phenotypes were more pronounced in diploids than in haploids, and also because diploids have a reduced tendency to acquire suppressor mutations.

**Table 1:** Strains and relevant genotypes.

Strain	Relevant genotype	Reference or source
DL3158	*MAT* **a** S288c *hog1*Δ::*KanMX*	Research Genetics
DL3181	*MAT* **a**/*MAT*α S288c *rgc2*Δ(*ask10*)::*KanMX/rgc2*Δ::*KanMX*	This study
DL3187	*MAT* **a** S288c (BY4742) *his3*Δ *leu2*Δ *ura3*Δ *lys2*Δ	Research Genetics
DL3193	*MAT* **a**/*MAT*α S288c *his3*Δ *leu2*Δ *ura3*Δ *lys2*Δ	Research Genetics
DL3203	*MAT* **a**/*MAT*α S288c *rgc1*Δ::*KanMX*/*rgc1*Δ::*KanMX*	This study
DL3207	*MAT* **a** S288c *rgc1*Δ::*KanMX rgc2*Δ::*KanMX*	This study
DL3209	*MAT* **a**/*MAT*α S288c *rgc1*Δ::*KanMX*/*rgc1*Δ::*KanMX rgc2*Δ::*KanMX*/*rgc2*Δ::*KanMX*	This study
DL3219	*MAT* **a** S288c *hog1*Δ::*KanMX rgc1*Δ::*KanMX rgc2*Δ::*KanMX*	This study
DL3226	*MAT* **a** S288c *fps1*Δ::*KanMX*	Research Genetics
DL3234	*MAT* **a**/*MAT*α S288c *fps1*Δ::*KanMX*/*fps1*Δ::*KanMX*	This study
DL3237	*MAT* **a**/*MAT*α S288c *gpd1*Δ::*KanMX*/*gpd1*Δ::*KanMX rgc1*Δ::*KanMX*/*rgc1*Δ::*KanMX rgc2*Δ::*KanMX*/*rgc2*Δ::*KanMX*	This study
DL3245	*MAT* **a**/*MAT*α S288c *fps1*Δ::*KanMX*/*fps1*Δ::*KanMX rgc1*Δ::*KanMX*/*rgc1*Δ::*KanMX rgc2*Δ::*KanMX*/*rgc2*Δ::*KanMX*	This study
DL3246	*MAT* **a** S288c *gpd1*Δ::*KanMX gpd2*Δ::*KanMX rgc1*Δ::*KanMX rgc2*Δ::*KanMX*	This study
DL3251	*MAT* **a**/*MAT*α S288c *gpd1*Δ::*KanMX*/*gpd1*Δ::*KanMX gpd2*Δ::*KanMX*/*gpd2*Δ::*KanMX rgc1*Δ::*KanMX*/*rgc1*Δ::*KanMX rgc2*Δ::*KanMX*/*rgc2*Δ::*KanMX*	This study
DL3254	*MAT* **a**/*MAT*α S288c *gpd2*Δ::*KanMX*/*gpd2*Δ::*KanMX rgc1*Δ::*KanMX*/*rgc1*Δ::*KanMX rgc2*Δ::*KanMX*/*rgc2*Δ::*KanMX*	This study
DL3947	*MAT* **a** S288c *hog1*Δ::*KanMX ste11*Δ::*Hph*	This study

### Suppressor screen

Three different genomic clones of *FPS1* were isolated from a high-copy genomic library in pRS202 (gift of P. Hieter) as suppressors of the temperature-sensitivity of a *rgc1/2*Δ mutant. The screen was conducted in the *rgc1/2*Δ mutant (DL3209) by plating transformations directly at 37°C. Plasmids were isolated from colonies arising after 3 days. A total of approximately 10,000 transformants were subjected to selection (as judged by low-temperature plating). This was calculated, based on an average insert size of 6 kb, to be approximately 5 genome-equivalents. Deletion analysis of one of these plasmids (p2165) confirmed that *FPS1* was responsible for the suppression activity.

### Plasmids

Two reporter plasmids for different transcriptional outputs were used in this study. One reporter, *PRM5* (−994 to +1)*-lacZ* (p1366) responds to the cell wall stress transcription factor, Rlm1 [23]. The other, *FPS1* (−933 to −57)-CYC1*-lacZ* (p2213), was constructed by PCR amplification of the 5′ non-coding region of *FPS1* using primers with Xho1 (upstream primer) and Sph1 (downstream primer) sites for cloning into the Xho1 and Sph1 sites of pLG178 (p904) [34]. This placed the regulatory sequences for *FPS1* upstream of the basal *CYC1* promoter linked to *lacZ*.

The entire *FPS1* gene was amplified by PCR from genomic yeast DNA (EG123 strain background) using a pair of primers 650 bp 5′ to the start codon and 500 bp 3′ to the stop codon. The primers were designed with a Not1 site (5′ primer) and a Sal1 site (3′ primer) for subcloning into pRS316 [35] to produce pRS316*-FPS1* (p2833). Open channel mutant *fps1-*Δ*1* in a multi-copy vector (YEp195-*fps1-*Δ*1*; p2496) was the gift of M. Mollapour. Open-channel mutant *fps1-C1* (YEp181-*fps1-C1-myc*; p2829) was the gift of S. Hohmann.

The *FPS1* gene, fused with a C-terminal Flag epitope, was expressed under the control of the *MET25* promoter. The *FPS1* coding sequence amplified from genomic DNA (EG123) with an XbaI site immediately 5′ to the initiation codon and a HindIII site immediately 3′ to the final codon and inserted into pRS426-*MET25^P^*-*FLAG* (p2186) so as to fuse the C-terminus with the Flag coding sequence, yielding *MET25^P^-FPS1-FLAG* (p2492). The YEpmyc181-*FPS1* plasmid (p2184) was the gift of S. Hohmann).

The *FPS1* gene was tagged at its C-terminus with tdTomato (red fluorescence) [36] and expressed under the control of its own promoter in two steps. First, the tdTomato coding sequence was subcloned from pRSET-B [*tdTomato*] (gift of R. Tsien) into pRS316 at the BamHI and EcoRI sites, yielding p2487. Next, the *FPS1* gene was amplified (omitting the endogenous stop codon) from genomic DNA (EG123) and inserted into p2487 using NotI and SpeI sites designed into the primers. This fused the *FPS1* reading frame with tdTomato, yielding pRS316-*FPS1-tdTomato* (p2489).

The *RGC2* gene was tagged at its C-terminus with 6xHis and expressed under the control of the *MET25* promoter. The *RGC2* coding sequence was amplified by PCR from genomic yeast DNA using primers that included XbaI and XhoI sites and cloned behind the *MET25* promoter in pUT36 (p2415) [37] to yield pUT36-*MET25^P^*-*RGC2-HIS_6_* (p2501). His-tagged C-terminal truncations of Rgc2 were also expressed under the control the *MET25* promoter. The first 1260 base pairs (amino acids 1–420) or 2160 base pairs (amino acids 1–720) of *RGC2* were amplified from genomic DNA (wild-type strain EG123) by PCR using a forward primer that contained an XbaI site immediately 5′ to the start codon and reverse primers that introduced a 6xHis tag followed by a stop codon and an XhoI site. The two regions were inserted into pUT36, resulting in pUT36-*MET25^P^-rgc2(1*–*420)-His_6_* (p2808) and pUT36-*MET25^P^-rgc2(1*–*720)-His_6_* (p2809).

The *RGC2* coding sequence was tagged at its C-terminus with two tandem copies of GFP and expressed under the control of the *MET25* promoter in three steps. In the first step, the *RGC2* promoter and coding sequence (omitting the endogenous stop codon) was amplified by PCR and inserted into the Not1 and Sma1 sites of pRS315[*GPF*] (p1164) [38] to yield pRS315-*RGC2-GFP* (p2478). In the second step, *RGC2-GFP* was amplified by PCR from p2478 and inserted in the same way into pRS315[*GFP*], to yield *RGC2-GFP_2_* (p2479). In the final step, the *RGC2-GFP_2_* coding sequence only was amplified by PCR and inserted into pRS414-*MET25^P^* (p976) using Spe1 and EcoRV sites designed into the primers. This yielded pRS414*-MET25^P^-RGC2-GFP_2_* (p2481).

The *RGC1* gene with 800 bp of upstream sequence was amplified by PCR from genomic EG123 DNA and using a forward primer that introduced a NotI site and a reverse primer that introduced a SalI site and cloned into centromeric vector pRS313 [35], yielding pRS313-*RGC1* (p2627). *FPS1* and *RGC1/2* constructs were validated by DNA sequence analysis and all were tested for functionality of these proteins by complementation of the cell lysis defects associated with an *fps1*Δ mutant or an *rgc1/2*Δ mutant, respectively.

### Measurements of Zymolyase sensitivity, cell wall stress reporter assays, intracellular glycerol concentrations, and glycerol efflux

Zymolyase sensitivity was carried out as described previously [39]. Promoter-*lacZ* expression experiments for determination of cell wall stress were carried out as described previously [40], with methods for β-galactosidase assays described in Zhao et al. [41]. Intracellular glycerol concentrations were measured in whole cells grown in YPD and centrifuged briefly to remove the culture supernatant. Enzymatic assays for glycerol were carried out using a kit from Boehringer Mannheim and normalized to A_600_ of the initial culture. Efflux measurements of ^14^C-glycerol were carried out as described by Tamas et al. [5]. Briefly, cells from log-phase cultures (30 ml) grown in YPD were washed in ice-cold MES buffer (10 mM MES, pH 6.0), resuspended in 1 ml ice-cold labeling buffer solution (10 mM MES buffer + 300 mM [^14^C]glycerol) and incubated for 1 hour at 30°C to load cells with labeled glycerol. Cells were then diluted 10-fold in ice-cold MES buffer to induce hypo-osmotic shock. Aliquots of cells were filtered onto Whatman GFB 25 mm discs at various time points, and washed with MES buffer. Radioactivity of dried filters was measured by a scintillation counter.

### Immunoblot detection of Mpk1, phospho-Mpk1, Fps1-Flag, Fps1-Myc, and Rgc2-His_6_

For detection of total Mpk1 and activated Mpk1, protein samples (20 µg) were separated by SDS-PAGE (7.5% gels) followed by immunoblot analysis. Total Mpk1 was detected with rabbit polyclonal antibodies from Santa Cruz Biotechnologies. Activated Mpk1 was detected with rabbit polyclonal α-phospho-p44/p42 MAPK (Thr^202^/Tyr^204^) antibodies (New England Biolabs). Both primary antibodies were used at a dilution of 1∶2000. Secondary donkey anti-rabbit antibodies (GE Healthcare) were used at a dilution of 1∶5000.

For detection of Fps1-Flag, protein samples (4 µg) were separated by SDS-PAGE (7.5% gels) followed by immunoblot analysis using mouse monoclonal α-FLAG antibody (M2; Sigma) at a dilution of 1∶10,000. For detection of Fps1-Myc, protein samples (25 µg) were separated by SDS-PAGE (7.5% gels) followed by immunoblot analysis using mouse monoclonal α-Myc antibody (9E10; BabCo) at a dilution of 1∶10,000. For detection of Rgc2-His_6_, protein samples (16 µg) were separated by SDS-PAGE (7.5% gels) followed by immunoblot analysis using mouse monoclonal α-tetra-HIS antibody (Qiagen) at a dilution of 1∶5000. Secondary antibodies (goat anti-mouse; Amersham) were used at a dilution of 1∶5000. For protein phosphatase treatment of Rgc2-His_6_, Nickel NTA agarose (Qiagen) was used to precipitate Rgc2-His_6_ from protein extracts (100 µg) prior to treatment with calf intestinal phosphatase (CIP; Promega) with, or without phosphatase inhibitor (10 mM Na_3_VO_4_) for 1 hour at 37°C. Precipitates were processed for immunoblot detection of Rgc2-His_6_.

### Fluorescence microscopic detection of Rgc2-GFP_2_ and Fps1-tdTomato

Diploid cells transformed with plasmids that express Rgc2-GFP_2_ with out without Fps1-tdTomato were grown in selective medium and visualized by fluorescence microscopy using a Zeiss Axioplan II with a 100x objective and fitted with a GFP and RFP filter. For hypo-osmotic shock experiments, log-phase cultures (1 ml) were centrifuged briefly to pellet cells, which were resuspended in 0.5 ml distilled water for 20 seconds to impose hypo-osmotic shock, followed by the addition of 0.5 ml 20 mM NaN_3_, 20 mM NaF, 20 mM Tris buffer to block further membrane transport [42] and set on ice for 20 seconds. Samples were centrifuged briefly to concentrate cells and mounted for microscopy. The membrane transport inhibitors were omitted from the time-course experiment.

## Supporting Information

Figure S1 Fps1 is stabilized in an *rgc1/2*Δ mutant, accounting for the higher protein levels in the mutant compared to WT. A His-tagged Fps1 construct (Open Biosystems ORF collection) was transformed into WT and *rgc1/2*Δ diploid cells (DL3193 and DL3209, respectively). Transformants were grown to mid-log phase in synthetic complete medium containing 2% raffinose, and Fps1 expression was induced with 4% galactose for 2 hours. Cells were washed in PBS and resuspended in synthetic complete medium containing 2% raffinose, 2% glucose, and 100 ug/mL cycloheximide. Samples were taken at the noted timepoints. Protein levels were normalized by Bradford assay.
(1.06 MB TIF)

Figure S2 Fps1-tdTomato localizes to punctate spots at the plasma membrane in the presence or absence of Rgc1/2. Fps1-tdTomato under the control of the endogenous Fps1 promoter (p2489) was transformed into WT and *rgc1/2*Δ diploid cells (DL3193 and DL3209, respectively) and visualized by fluorescence microscopy using a Zeiss Axioplan II with a 100× objective fitted with an RFP filter. There is no obvious change in the localization, number, or intensity of the Fps1 punctae in the*rgc1/2*Δ mutant, as compared to WT.
(6.41 MB TIF)

Figure S3 The Hog1-independent Rgc2 phosphorylation induced by hyper-osmotic stress is not the result of crosstalk from the mating pathway. The genomic copy of *STE11* was deleted from a *hog1*Δ strain (DL3158) using a PCR-amplified Hph (encoding hygromycin B resistance) cassette with 50 nucleotides of *STE11* non-coding sequence at each end. The deletion was confirmed by colony PCR at both ends of the replacement cassette. The resulting *hog1*Δ::*KanMX ste11*Δ::*Hph* strain (DL3947) and DL3158 were transformed with a plasmid that expresses Gca2-His_6_ (p2501). Transformants were grown to mid-log phase and exposed to hyper-osmotic (1.8M sorbitol) shock for 1 minute. Protein extracts were prepared and separated by SDS-PAGE for immunoblot detection of Gca2-His_6_.
(1.58 MB TIF)

Figure S4 Phosphorylation of Rgc2 in response to oxidative stress occurs independently of the cell wall integrity MAP kinase pathway. The indicated strains in the BY4741 genetic background (Research Genetics) were transformed with a plasmid expressing Ask10-HA (pAK3, gift of R. Strich). Transformants were grown to mid-log phase and treated for 30 min with 0.4 mM H_2_O_2_, as described previously (Cohen et al., 2003). Extracts were prepared and separated by SDS-PAGE for immunoblot detection of Ask10-HA.
(2.41 MB TIF)

## References

[1] Nevoigt E, Stahl U (1997). Osmoregulation and glycerol metabolism in the yeast *Saccharomyces cerevisiae*.. FEMS Micro Rev.

[2] Kayingo G, Wong B (2005). The MAP kinase Hog1p differentially regulates stress-induced production and accumulation of glycerol and D-arabitol in *Candida albicans*.. Microbiology.

[3] Luyten K, Albertyn J, Skibbe WF, Prior BA, Ramos J (1995). Fps1, a yeast member of the MIP family of channel proteins, is a facilitator for glycerol uptake and efflux and is inactive under osmotic stress.. EMBO J.

[4] Sutherland FCW, Lages F, Lucas C, Luyten K, Albertyn J (1997). Characteristics of Fps1-dependent and –independent glycerol transport in *Saccharomyces cerevisiae*.. J Bacteriol.

[5] Tamas MJ, Luyten K, Sutherland FCW, Hernandez A, Albertyn J (1999). Fps1p controls the accumulation and release of the compatible solute glycerol in yeast osmoregulation.. Molec Micro.

[6] Philips J, Herskowitz I (1997). Osmotic balance regulates cell fusion during mating in *Saccharomyces cerevisiae*.. J Cell Biol.

[7] Hohmann S (2002). Osmotic stress signaling and osmoadaptation in yeasts.. Microbiol Mol Biol Rev.

[8] Hohmann S, Krantz M, Norlander B (2007). Yeast osmoregulation.. Meth Enzymol.

[9] Mollapour M, Piper PW (2007). Hog1 mitogen-activated protein kinase phosphorylation targets the yeast Fps1 aquaglyceroporin for endocytosis, thereby rendering cells resistant to acetic acid.. Mol Cell Biol.

[10] Wysocki R, Chery CC, Wawrzycka D, Van Hulle M, Cornelis R (2001). The glycerol channel Fps1p mediates uptake of arsenite and antimonite in *Saccharomyces cerevisiae*.. Molec Micro.

[11] Thorsen M, Di Y, Tangemo C, Morillas M, Ahmadpour D, Van der Does C (2006). The MAPK Hog1p modulates Fps1p-dependent arsenite uptake and tolerance in yeast.. Mol Biol Cell.

[12] Fadri M, Daquinag A, Wang S, Xue T, Kunz J (2005). The pleckstrin homology domain proteins Slm1 and Slm2 are required for actin cytoskeleton organization in yeast and bind phosphatidylinositol-4,5,-bisphosphate and TORC2.. Mol Biol Cell.

[13] Yu JW, Mendrola JM, Audhya A, Singh S, Keleti D (2004). Genome-wide analysis of membrane targeting by *S. cerevisiae* pleckstrin homology domains.. Mol Cell.

[14] van Rossum DB, Patterson RL, Sharma S, Barrow RK, Kornberg M (2005). Phospholipase Cg1 controls surface expression of TRPC3 through an intermolecular PH domain.. Nature.

[15] Page N, Sheraton J, Brown JL, Stewart RC, Bussey H (1996). Identification of *ASKl0* as a multicopy activator of Skn7p-dependent transcription of a *HIS3* reporter gene.. Yeast.

[16] Brown JL, North S, Bussey H (1993). *SKN7*, a yeast multicopy suppressor of a mutation affecting cell wall β-glucan assembly, encodes a product with domains homologous to prokaryotic two-component regulators and to heat shock transcription factors.. J Bacteriol.

[17] Alberts AS, Bouquin N, Johnston LH, Treisman R (1998). Analysis of RhoA-binding proteins reveals an interaction domain conserved in heterotrimeric G protein beta subunits and the yeast response regulator protein Skn7.. J Biol Chem.

[18] Ketela T, Green R, Bussey H (1999). *Saccharomyces cerevisiae* Mid2p is a potential cell wall stress sensor and upstream activator of the *PKC1-MPK1* cell integrity pathway.. J Bacteriol.

[19] Li S, Dean S, Li Z, Horecka J, Deschenes RJ (2002). The eukaryotic two-component histidine kinase Sln1p regulates *OCH1* via the transcription factor, Skn7p.. Mol Biol Cell.

[20] Cohen TJ, Lee K, Rutkowski LH, Strich R (2003). Ask10p meadiates the oxidative stress-induced destruction of the *Saccharomyces cerevisiae* C-type cyclin Ume3p/Srb11p.. Euk Cell.

[21] Cooper KF, Mallory MJ, Smith JS, Strich R (1997). Stress and developmental regulation of the yeast C-type cyclin *UME3* (*SRB11/SSN8*).. EMBO J.

[22] Hengartner CJ, Myer VE, Liao S-M, Wilson CJ, Koh SS (1998). Temporal regulation of RNA polymerase II by Srb10 and Kin28 cyclin-dependent kinases.. Mol Cell.

[23] Jung US, Sobering AK, Romeo MJ, Levin DE (2002). Regulation of the yeast Rlm1 transcription factor by the Mpk1 cell wall integrity MAP kinase.. Molec Micro.

[24] Kurtz MB, Douglas CM (1994). Lipopeptide inhibitors of fungal glucan synthase.. J Med Vet Mycol.

[25] Larsson K, Ansell R, Eriksson P, Adler L (1993). A gene encoding sn-glycerol 3-phosphate dehydrogenase (NAD+) complements an osmosensitive mutant of Saccharomyces cerevisiae.. Mol Microbiol.

[26] Albertyn J, Hohmann S, Thevelein JM, Prior BA (1994). GPD1, which encodes glycerol-3-phosphate dehydrogenase, is essential for growth under osmotic stress in Saccharomyces cerevisiae, and its expression is regulated by the high-osmolarity glycerol response pathway.. Mol Cell Biol.

[27] Remize F, Barnavon L, Dequin S (2001). Glycerol export and glycerol-3-phosphate dehydrogenase, but not glycerol phosphatase, are rate limiting for glycerol production in *Saccharomyces cerevisiae*.. Metabol Eng.

[28] Huh WK, Falvo JV, Gerk LC, Carroll AS, Howson RW (2003). Global analysis of protein localization in budding yeast.. Nature.

[29] Tamas MJ, Karlgren S, Bill RM, Hedfalk K, Allegri L (2003). A short regulatory domain restricts glycerol transport through yeast Fps1p.. J Biol Chem.

[30] Hedfalk RM, Bill J, Mullins GL, Karlgren S, Filipsson C (2004). A regulatory domain in the C-terminal extension of the yeast glycerol channel Fps1p.. J Biol Chem.

[31] O'Rourke SM, Herskowitz I (1998). The Hog1 MAPK prevents cross talk between the HOG and pheromone response MAPK pathways in *Saccharomyces cerevisiae*.. Genes Dev.

[32] Bodenmiller B, Campbell D, Gerrits B, Lam H, Jovanovic M (2008). PhosphoPep—a database of protein phosphorylation sites in model organisms.. Nature Biotechnology.

[33] Albuquerque CP, Smolka MB, Payne SH, Bafna V, Eng J (2008). A multidimensional chromatography technology for in-depth phosphoproteome analysis.. Mol Cell Proteomics.

[34] Guarente L, Mason T (1983). Heme regulates transcription of the *CYC1* gene of *S. cerevisiae* via an upstream activation site.. Cell.

[35] Sikorski RS, Hieter P (1989). A system of shuttle vectors and yeast host strains designed for efficient manipulation of DNA in *Saccharomyces cerevisiae*.. Genetics.

[36] Shaner NC, Campbell RE, Steinbach PA, Giepmans BNG, Palmer AE (2004). Improved monomeric red, orange and yellow fluorescent proteins derived from *Discoma* sp. red fluorescent protein.. Nat Biotech.

[37] Millson SH, Truman AW, King V, Prodromou C, Pearl LH (2005). A two-hybrid screen of the yeast proteome for Hsp90 interactors uncovers a novel Hsp90 chaperone requirement in the activity of a stress-activated mitogen-activated protein kinase, Slt2p (Mpk1p).. Euk Cell.

[38] Rajavel M, Philip B, Buehrer BM, Errede B, Levin DE (1999). Mid2 is a putative sensor for cell integrity signaling in *Saccharomyces cerevisiae*.. Mol Cell Biol.

[39] Newman HA, Romeo MJ, Lewis SE, Yan BC, Orlean P (2005). Gpi19, the *Saccharomycas cerevisiae* homologue of mammalian PIG-P, is a subunit of the intial enzyme for glycosylphosphatidylinositol anchor biosynthesis.. Euk Cell.

[40] Kim K-Y, Truman AW, Levin DE (2008). Yeast Mpk1 mitogen-activated protein kinase activates transcription through Swi4/Swi6 by a noncatalytic mechanism that requires upstream signal.. Mol Cell Biol.

[41] Zhao C, Jung US, Garrett-Engele P, Roe T, Cyert MS (1998). Temperature-induced expression of yeast *FKS2* is under the dual control of protein kinase C and calcineurin.. Mol Cell Biol.

[42] Yeo SCL, Xu L, Ren J, Boulton VJ, Wagle MD (2003). Vps20p and Vta1p interact with Vps4p and function in multivesicular body sorting and endosomal transport in *Saccharomyces cerevisiae*.. J Cell Sci.

