# Supplementary figures and images for: Identification of Positive Regulators of the Yeast Fps1 Glycerol Channel
Source: PLoS Genet. 2009 Nov 26;5(11):e1000738. doi: 10.1371/journal.pgen.1000738 (PMC2773846; doi:10.1371/journal.pgen.1000738)

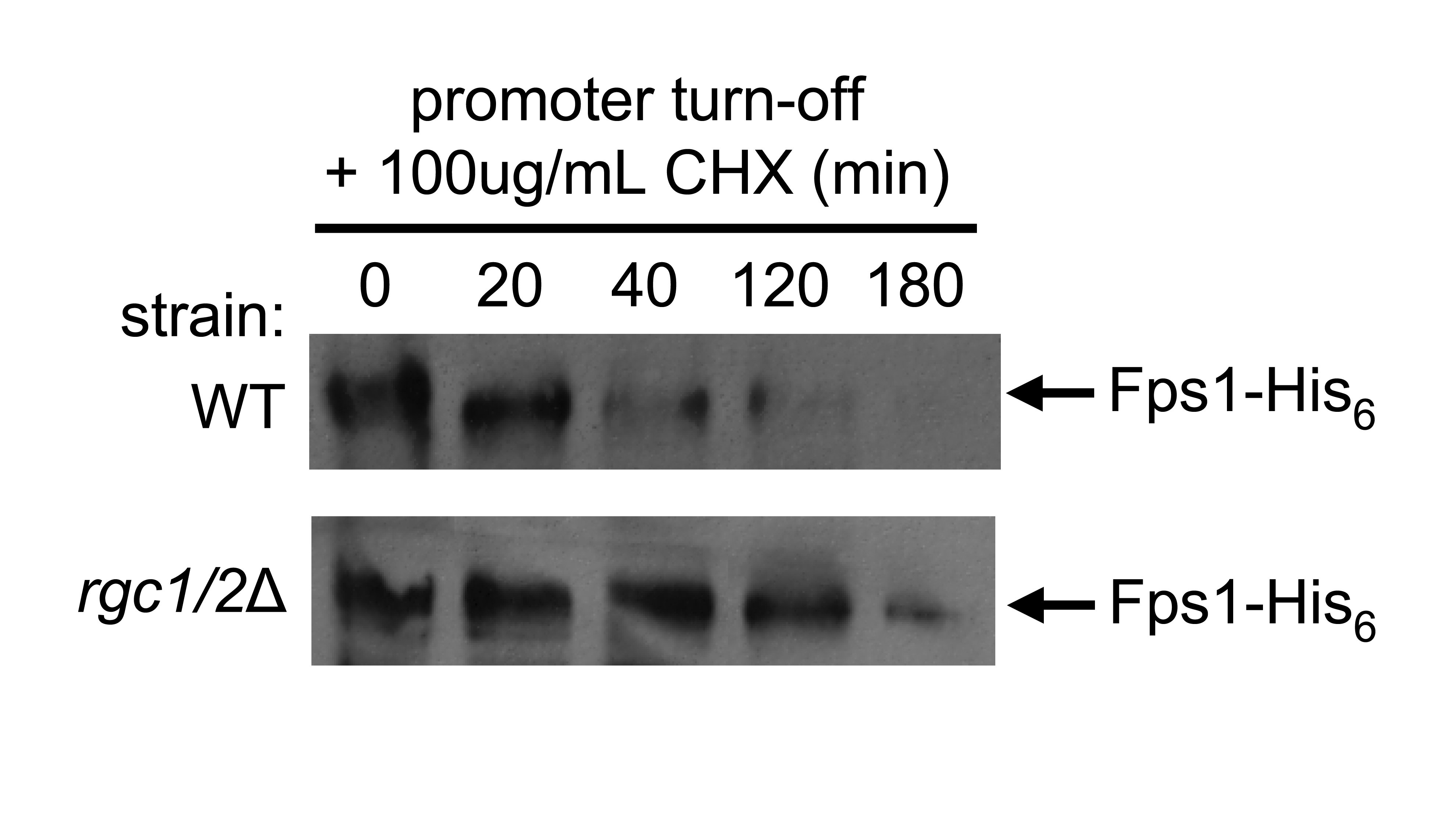

Supplement: Figure S1 — Fps1 is stabilized in an rgc1/2Δ mutant, accounting for the higher protein levels in the mutant compared to WT. A His-tagged Fps1 construct (Open Biosystems ORF collection) was transformed into WT and rgc1/2Δ diploid cells (DL3193 and DL3209, respectively). Transformants were grown to mid-log phase in synthetic complete medium containing 2% raffinose, and Fps1 expression was induced with 4% galactose for 2 hours. Cells were washed in PBS and resuspended in synthetic complete medium containing 2% raffinose, 2% glucose, and 100 ug/mL cycloheximide. Samples were taken at the noted timepoints. Protein levels were normalized by Bradford assay. (1.06 MB TIF) [file pgen.1000738.s001.tif]

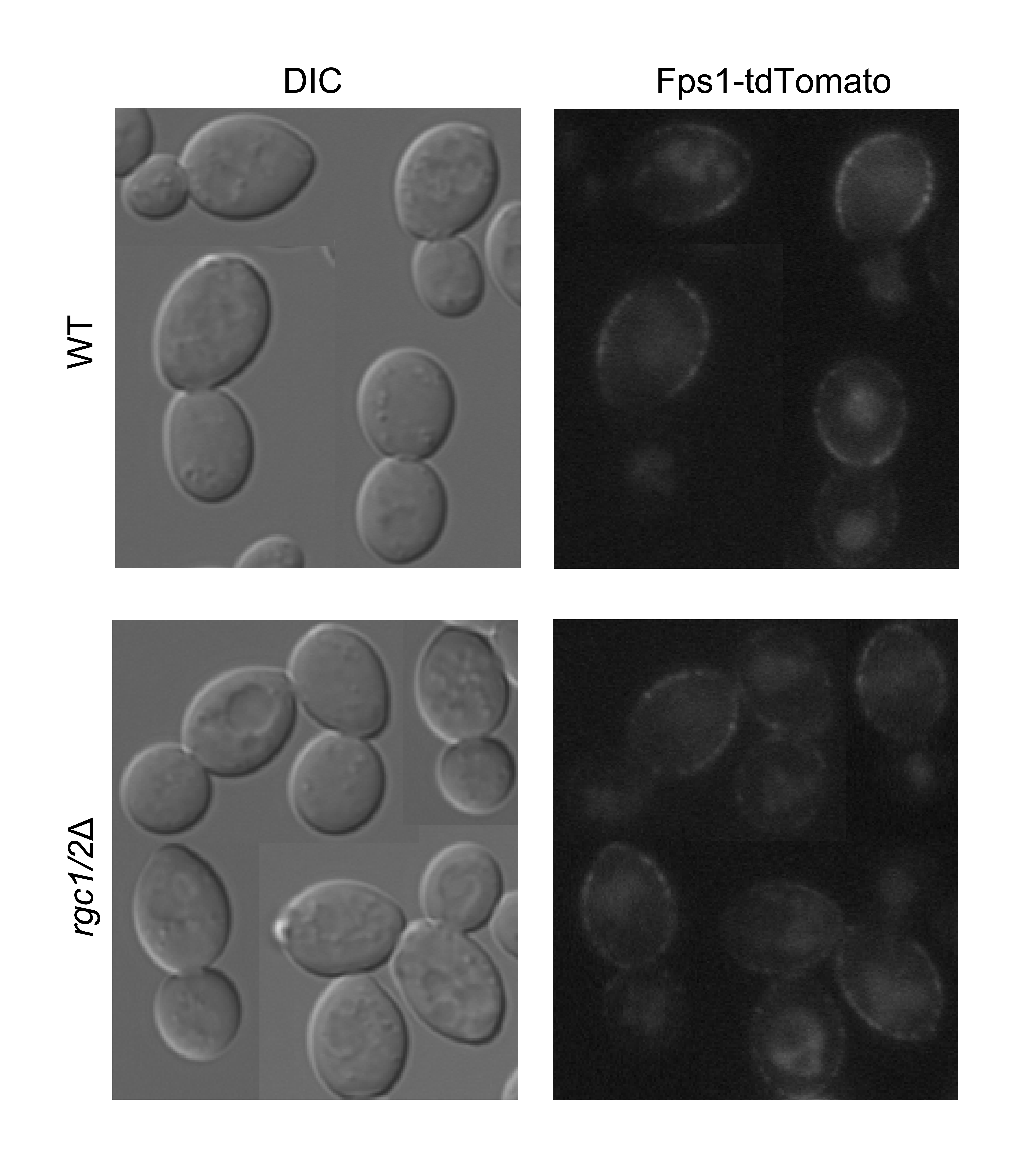

Supplement: Figure S2 — Fps1-tdTomato localizes to punctate spots at the plasma membrane in the presence or absence of Rgc1/2. Fps1-tdTomato under the control of the endogenous Fps1 promoter (p2489) was transformed into WT and rgc1/2Δ diploid cells (DL3193 and DL3209, respectively) and visualized by fluorescence microscopy using a Zeiss Axioplan II with a 100× objective fitted with an RFP filter. There is no obvious change in the localization, number, or intensity of the Fps1 punctae in thergc1/2Δ mutant, as compared to WT. (6.41 MB TIF) [file pgen.1000738.s002.tif]

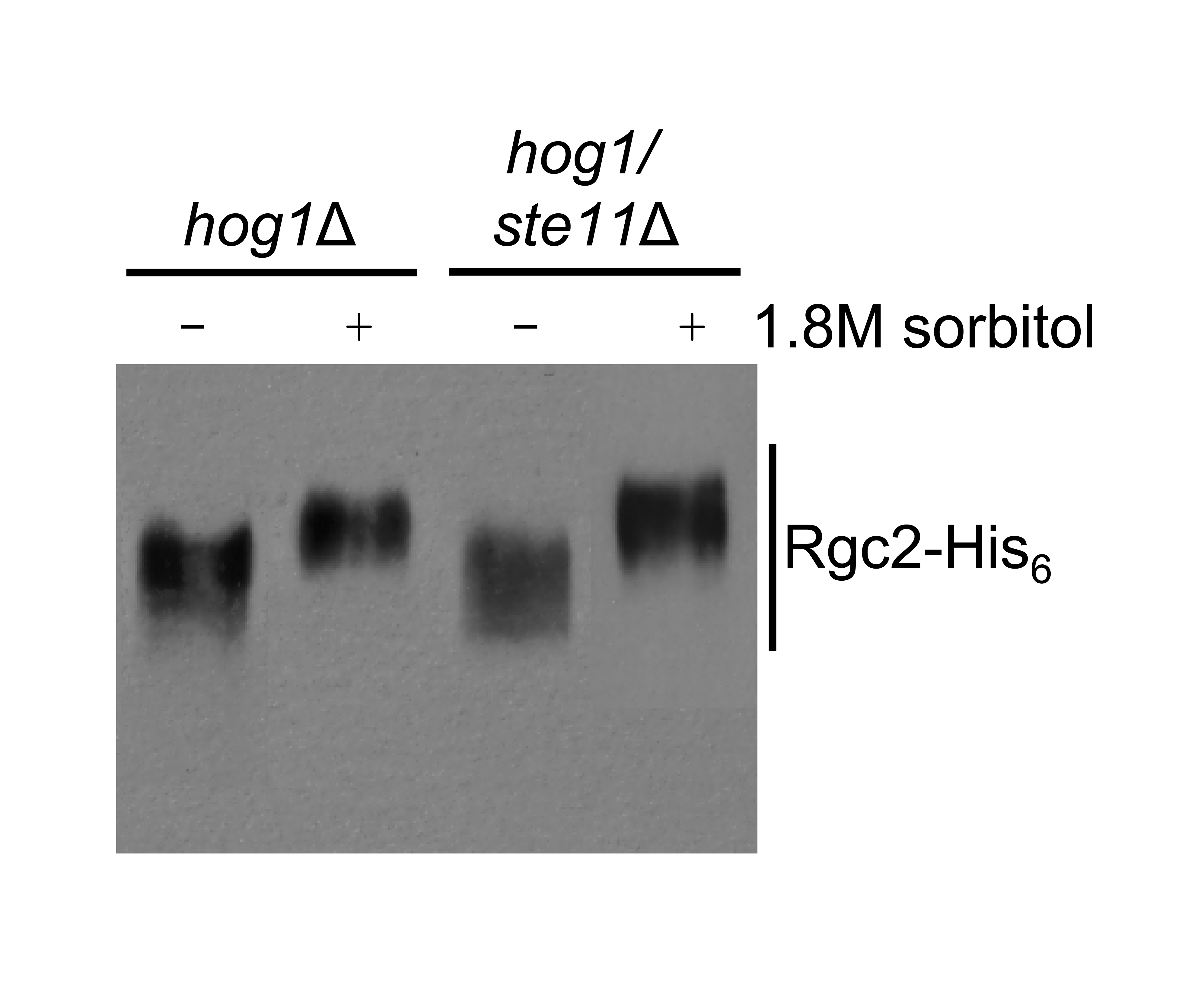

Supplement: Figure S3 — The Hog1-independent Rgc2 phosphorylation induced by hyper-osmotic stress is not the result of crosstalk from the mating pathway. The genomic copy of STE11 was deleted from a hog1Δ strain (DL3158) using a PCR-amplified Hph (encoding hygromycin B resistance) cassette with 50 nucleotides of STE11 non-coding sequence at each end. The deletion was confirmed by colony PCR at both ends of the replacement cassette. The resulting hog1Δ::KanMX ste11Δ::Hph strain (DL3947) and DL3158 were transformed with a plasmid that expresses Gca2-His6 (p2501). Transformants were grown to mid-log phase and exposed to hyper-osmotic (1.8M sorbitol) shock for 1 minute. Protein extracts were prepared and separated by SDS-PAGE for immunoblot detection of Gca2-His6. (1.58 MB TIF) [file pgen.1000738.s003.tif]

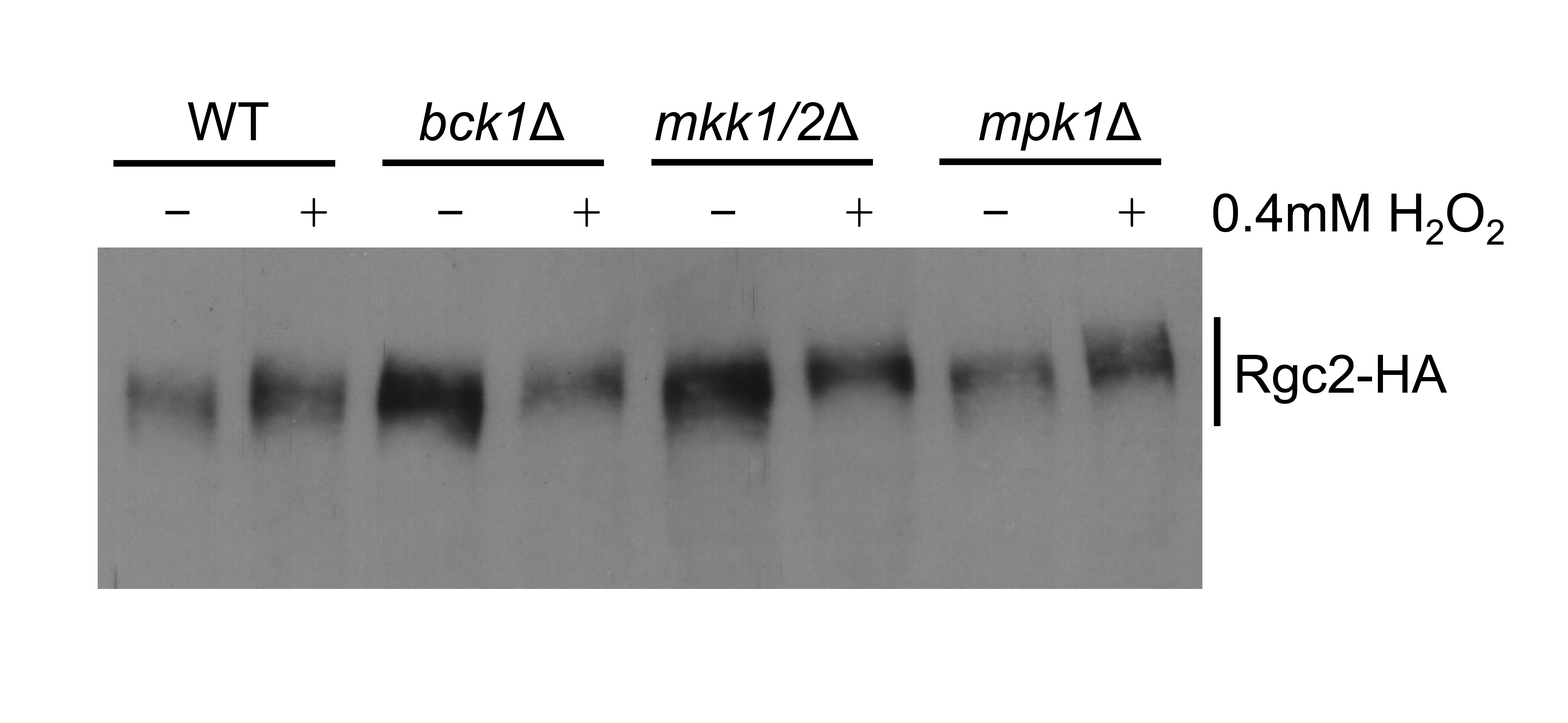

Supplement: Figure S4 — Phosphorylation of Rgc2 in response to oxidative stress occurs independently of the cell wall integrity MAP kinase pathway. The indicated strains in the BY4741 genetic background (Research Genetics) were transformed with a plasmid expressing Ask10-HA (pAK3, gift of R. Strich). Transformants were grown to mid-log phase and treated for 30 min with 0.4 mM H2O2, as described previously (Cohen et al., 2003). Extracts were prepared and separated by SDS-PAGE for immunoblot detection of Ask10-HA. (2.41 MB TIF) [file pgen.1000738.s004.tif]
